# Study protocol for a randomized controlled trial for anterior inguinal hernia repair: transrectus sheath preperitoneal mesh repair compared to transinguinal preperitoneal procedure

**DOI:** 10.1186/1745-6215-14-65

**Published:** 2013-03-03

**Authors:** M Wiesje Prins, Giel G Koning, Eric F Keus, Patrick WHE Vriens, Roland MHG Mollen, Willem L Akkersdijk, Cees JHM van Laarhoven

**Affiliations:** 1 Department of Surgery, Radboud University Nijmegen Medical Centre, Geert Grooteplein Zuid 10, 6525 GA, Nijmegen, Gelderland, The Netherlands; 2 St Elisabeth Hospital, Hilvarenbeekse Weg 60, 5022GC, Tilburg, The Netherlands; 3 TweeSteden Hospital, Kasteellaan 2, 5141 BM, Waalwijk, The Netherlands; 4 Gelderse Vallei Hospital, Willy Brandtlaan 10, 6716 RP, Ede, The Netherlands; 5 St Jansdal Hospital, Wethouder Jansenlaan 90, 3844 DG, Harderwijk, The Netherlands

**Keywords:** Chronic pain, Inguinal, Hernia, Preperitoneal, Mesh, TREPP, TIPP, Open repair, Trial, Randomized

## Abstract

**Background:**

Anterior open treatment of the inguinal hernia with a tension-free mesh has reduced the incidence of hernia recurrence. The Lichtenstein procedure is the current reference technique for inguinal hernia treatment. Chronic pain has become the main postoperative complication after surgical inguinal hernia repair, especially following Lichtenstein. Preliminary experiences with a soft mesh positioned in the preperitoneal space (PPS) by transinguinal preperitoneal (TIPP) or total extraperitoneal (TEP) technique, showed promising results considering the reduction of postoperative chronic pain. Evolution of surgical innovations for inguinal hernia repair led to an open, direct approach with preperitoneal mesh position, such as TIPP. Based on the TIPP procedure, another preperitoneal repair has been recently developed, the transrectus sheath preperitoneal (TREPP) mesh repair.

**Methods:**

The ENTREPPMENT trial is a multicentre randomized clinical trial. Patients will be randomly allocated to anterior inguinal hernia repair according to the TREPP mesh repair or TIPP procedure. All patients with a primary unilateral inguinal hernia, eligible for operation, will be invited to participate in the trial. The primary outcome measure will be the number of patients with postoperative chronic pain. Secondary outcome measures will be serious adverse events (SAEs), including recurrence, hemorrhage, return to daily activities (for example work), operative time and hospital stay. Alongside the trial health status, an economic evaluation will be performed. To demonstrate that inguinal hernia repair according to the TREPP technique reduces the percentage of patients with postoperative chronic pain from 12% to <6%, a sample size of 800 patients is required (two-sided test, α = 0.05, 80% power).The ENTREPPMENT trial aims to evaluate the TREPP and TIPP procedures from patients’ perspective. It is hypothesized that the TREPP technique may reduce the number of patients with any form of postoperative chronic pain by 50% compared to the TIPP procedure.

**Trial registration:**

Current Controlled Trials: ISRCTN18591339

## Background

An inguinal hernia has been reported as a common surgical problem. In The Netherlands, approximately 30,000 inguinal hernia repairs are performed each year [1]. Tension-free mesh repair has reduced the incidence of recurrence (<5%) [2]. However, postoperative chronic pain after inguinal hernia repair is the main complication, especially following Lichtenstein procedure [3-5]. Lichtenstein is the current reference technique for inguinal hernia repair [6]. The incidence of chronic pain and (hypothetical) pathophysiological mechanisms of chronic pain have been reported. Studies have shown possible advantages in preperitoneal mesh positioning, due to the lack of need for fixating the mesh. Furthermore, the approach in which the inguinal region with its nerves is completely avoided during dissection resulted in less patients with postoperative chronic pain [7,8]. The effect of the preperitoneal mesh position is in line with those reported after total extraperitoneal (TEP) and transabdominal preperitoneal (TAPP) procedures.

The transinguinal preperitoneal (TIPP) procedure has been described as an alternative open preperitoneal mesh repair [9,10]. Results of the TULIP trial, comparing TIPP with the Lichtenstein procedure, concluded significantly less patients with postoperative chronic pain after TIPP in the first postoperative year [11]. Based on the TULIP trial results and the low risk of bias methodology, the TIPP procedure will be used as the control intervention in this trial.

Based on the TIPP procedure, another open preperitoneal mesh repair has been developed, the transrectus sheath preperitoneal (TREPP) mesh repair. Description of the technique and results of the first 50 TREPP cases have been reported [12]. This pilot study showed promising results for TREPP. The TREPP principles are: an open technique, easy to learn, sutureless preperitoneal mesh position, and avoiding the inguinal canal and inguinal nerves peroperatively. These principles are in concordance with the reported recommendations of Reinpold [13].

The aim of this randomized clinical trial is to evaluate the TREPP and TIPP procedures. The trial design focuses on the three dimensions of possible errors: bias, the ‘play of chance’ and the chosen outcome [14-16].

## Methods

Prior to the start of the trial, the study protocol was written and will be published. The ENTREPPMENT trial is registered with the International Standard Randomised Controlled Trial Number (ISRCTN) Register ( http://controlled-trials.com/ISRCTN18591339). The protocol was ethically approved by the official Independent Review Board Nijmegen (2012/060) and registered nationally (NL38842.091.12) [17].

### Design

The ENTREPPMENT trial will be a prospective randomized multicentre trial. Two inguinal hernia repair techniques, TREPP and TIPP, will be compared.

Patients will be included at the outpatient clinics of the participating centers (Radboud University Nijmegen Medical Centre, Nijmegen; St Elisabeth Hospital, Tilburg; TweeSteden Hospital, Tilburg/Waalwijk; Gelderse Vallei Hospital, Ede; and St Jansdal Hospital, Harderwijk) by surgeons and supervised residents.

Prior to the start of the trial, group sessions in the operation room (and by discussion) for complete standardization and uniformity of the TREPP and TIPP procedures will be performed with the participating surgeons. All surgeons will be assessed by an experienced colleague (proctor) prior to their participation in the trial. The proctors will decide whether the surgeon has completed the learning curves of both techniques and can start the procedures in the trial.

Randomized patients will be operated on according to this protocol. Dedicated hernia surgeons will perform the operations or will supervise the residents. The same mesh with memory ring will be used in both techniques (Polysoft 16 × 9.5cm; Bard, Benelux, Belgium). The skin will be closed intracutaneously.

### Patients

Patients with a primary unilateral inguinal hernia, visiting the outpatient clinics at the participating centers will be invited to participate.

Inclusion criteria are: primary unilateral groin hernia, aged between 18 and 80 years, American Society of Anesthesiologists’ (ASA) classification 1 to 3, and signed informed consent.

Exclusion criteria are: recurrent inguinal hernia, scrotal hernia, femoral hernia, acute incarcerated inguinal hernia, psychiatric disease, or other reasons making follow-up or questionnaires unreliable, and previous preperitoneal surgery, for example radical prostatectomy.

### Intervention

The TREPP technique has been described [12]. To reach the preperitoneal space (PPS), a 5 cm transverse incision is made approximately 1 cm cranial to the pubic bone. The anterior rectus sheath is opened by transverse incision. After retraction of the muscle fibers medially, the inferior epigastric vein and artery are identified and retracted medially. The underlying transverse fascia is opened transversely as well. With a gentle movement, the PPS is dissected and a medial hernia may be reduced immediately. Using the iliac vessels as a landmark, the funiculus is identified with the spermatic cord, the testicular vessels and a possible lateral hernia. The latter (if present) may now be reduced. Using three long and thin retractors, a perfect overview of the PPS may be achieved and all possible hernia orifices (medial, lateral and/or femoral) can be visualized. The soft mesh is positioned in the PPS and covers the complete myopectineal orifice of Fruchaud. After deployment, the abdominal pressure keeps the mesh in position without necessitating any fixation. The anterior rectus sheath and the fascia of Scarpa are closed with Vicryl (Ethicon, Livingston, UK).

### Control intervention

The TIPP procedure has been described by Pelissier [9,10] and evaluated in the TULIP trial [18]. In brief, using the transinguinal approach, nerves will be identified and spared. The hernia sac is reduced into the PPS. The PPS is dissected bluntly by a finger. A soft mesh with memory ring is positioned in the PPS without the need for fixation.

### Outcome measures

The primary outcome measure will be the number of patients with postoperative chronic pain. Chronic pain is defined by the International Association for the Study of Pain as any visual analog scale (VAS) score above zero that lasts for more than 3 months postoperatively [19]. To prevent the disadvantages of a single measurement, postoperative chronic pain will be assessed at 6 and 12 months.

Secondary outcome measures are serious adverse events (SAEs) during the first year after randomization. SAEs are: death, irrespective of the cause; life-threatening event; re-admission to the hospital; hemorrhage; wound infection, either surgical site or deep infection; and recurrence (symptomatic) of the inguinal hernia. When in doubt considering the seriousness of an event, a blinded expert opinion will be obtained.

Other secondary outcome measures will be hospital stay, operative time, numbness and return to daily activities. Alongside the trial, an economic evaluation and the health status will be assessed.

### Anesthesia and analgesia

Preoperatively, all patients will visit the anesthesiologist. One analgesia protocol will be used in combination with a standardized regimen regarding both postoperative pain and nausea medication. These regimes are based on daily practice; the standardization serves to avoid unnecessary bias. First choice of anesthesia technique is spinal anesthesia, owing to its ease and expedited postoperative recovery, avoiding the risks of general anesthesia (nausea, tube, aspiration, heart/lung complications). If the patient declines, general anesthesia will be permitted. All anesthesiologists will be experienced with both general and spinal anesthesia. The wound will be infiltrated with 10 cc bupivacaine 0.1%.

### Randomization and blinding

The randomization will be stratified by centre. Correct generation of the allocation sequence, allocation concealment, blinding and follow-up will be warranted. The allocation sequence will be computer-generated. The trial office will be contacted online prior to incision. The nurse in the operating room will write down the technique for the surgeon, so the patient will be unaware of the technique used. Operation reports will be blinded in the electronic patient files and no access will be allowed for the outcome assessors. Plasters will be positioned identically after both techniques. During follow-up, questionnaires will be completed before contact with outcome assessors and physical examination, to provide optimal masking.

### Unblinding protocol

Patients will be informed on request about the performed procedure only after completing the last follow-up visit.

### Data recording and follow-up

**Figure 1:**
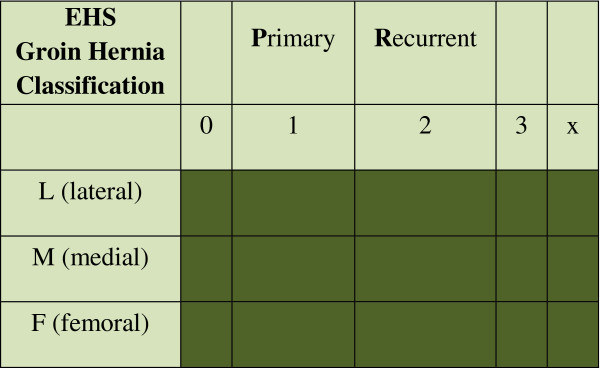
**European Hernia Society (EHS) groin hernia classification [**[20]**].**

All hernias will be classified according to the European Hernia Society (EHS) hernia classification (Figure 1) [20].

All included patients will be interviewed preoperatively according to the Pain Disability Index (PDI), Short Form 36 (SF-36) and EQ-5D. Preoperative pain scores will be assessed by the Verbal Rating Scale (VRS) for pain. All patients will keep a pain diary including the PDI and the VRS for pain during the first 14 postoperative days.

The pinprick-test will be used to assess numbness in the dermatomes related to the inguinal nerves on the operated side. A figure of dermatomes will be used for anatomical orientation [21].

Follow-up, including the three questionnaires and a physical examination, will be scheduled at 14 days, 6 months and 1 year postoperatively.

### Ethics and informed consent

This study is conducted in concordance with the principles of the Declaration of Helsinki [22] and Good Clinical Practice guidelines. The protocol was ethically approved by the official Independent Review Board Nijmegen (2012/060) and registered nationally (NL38842.091.12) [17]. A Data and Safety Monitoring Board (DSMB) is established to perform safety surveillance and to perform interim analyses on the safety data, as described.

### Analysis and sample size

It is hypothesized that the TREPP technique reduces the percentage of patients with any form of postoperative chronic pain from 12% to 6%, or less. The TULIP trial shows an incidence of patients with any form of postoperative chronic pain, either continuous or pain during activity, of 12%. Step-up studies show postoperative chronic pain after the TREPP technique, self-monitored by patients and measured by a non-validated questionnaire, of 7% in a large undefined group.

Based on an absolute risk reduction from 12% to 6%, a sample size of 720 patients is required (two-sided test, α = 0.05, 80% power).

In order to compensate for possible loss to follow-up, 800 patients will be enrolled. The study end is expected 2.5 years after the start of the trial: 1.5 years of inclusion and 1 year of follow-up. No interim analyses will be planned.

### Statistics

The analysis will be performed on the basis of the intention-to-treat principle. Logistic regression with centre as cofactor will be used to analyze binary outcomes. Similarly, analysis of covariance with centre as factor will be used to analyze continuous outcomes. Skewed variables will be log transformed. A two-sided *P* value of <0.05 will be considered to be significant and 95% confidence intervals will be calculated.

### Reporting

**Figure 2:**
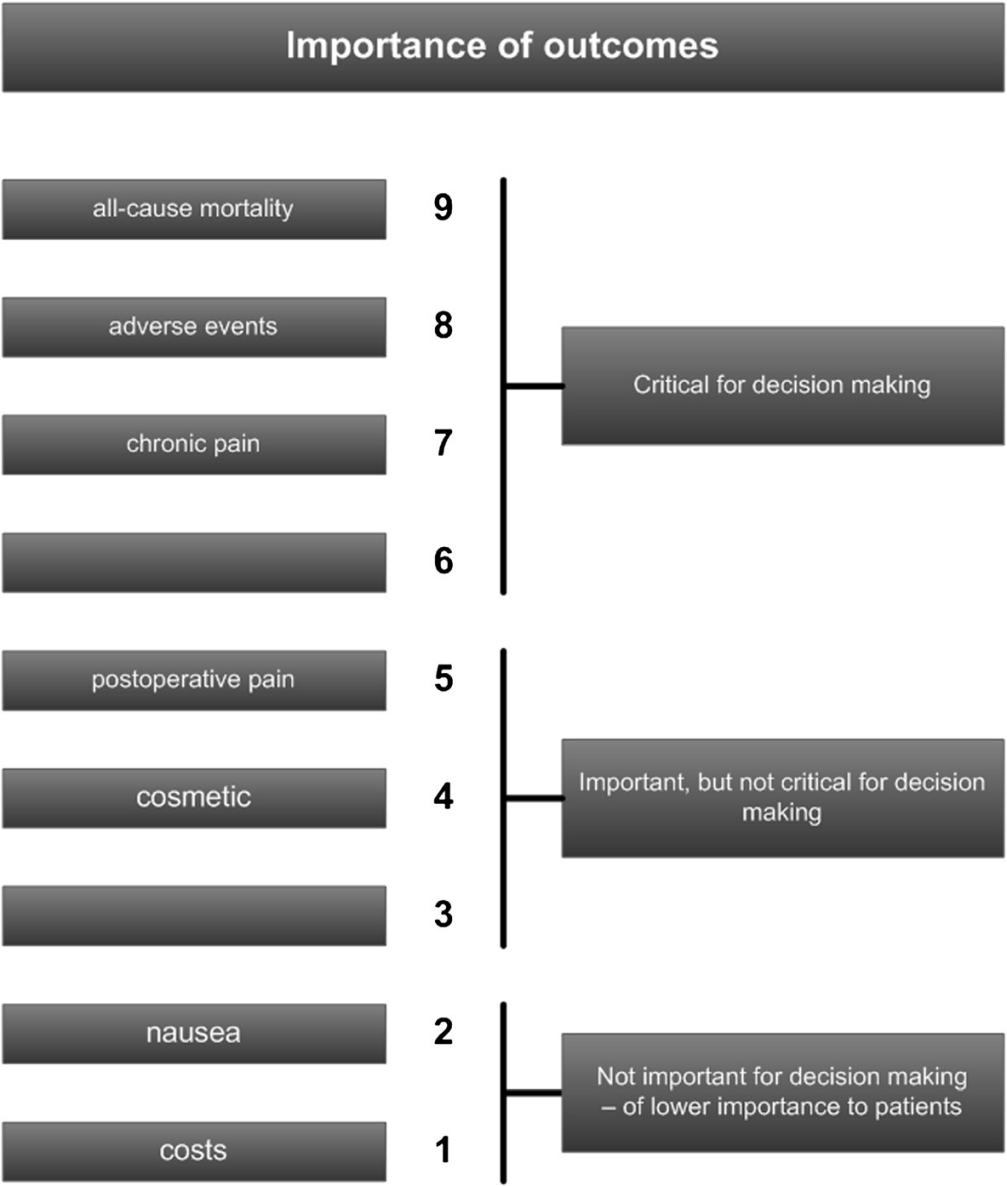
**Example of the importance of outcomes from patients’ perspective according to GRADE 2008 [**[15]**].**

The ENTREPPMENT trial findings will be graded to facilitate critical decision making from the patients’ perspective according to the Grading of Recommendations Assessment, Development and Evaluation (GRADE) Working Group (Figure 2) [15].

Study outcomes will be reported in concordance to the recently updated Consolidated Standards of Reporting Trials (CONSORT) checklist [23].

### Trial status

Approved by the official Independent Review Board Nijmegen (2012/060). Inclusion not started.

## Abbreviations

ASA: American Society of Anesthesiologists;CONSORT: Consolidated Standards of Reporting Trials;DSMB: Data and Safety Monitoring Board;EHS: European Hernia Society;GRADE: Grading of Recommendations Assessment Development and Evaluation;ISRCTN: International Standard Randomised Controlled Trial Number;PDI: Pain Disability Index;PPS: Preperitoneal space;SAE: Serious adverse event;SF-36: Short Form 36;TAPP: Transabdominal preperitoneal;TEP: Total extraperitoneal;TIPP: Transinguinal preperitoneal;TREPP: Transrectus sheath preperitoneal;VAS: Visual analog scale;VRS: Verbal Rating Scale

## Competing interests

The authors declare that they have no competing interests.

## Authors’ contributions

MP, GK and FK designed the trial and wrote the manuscript. GK, PV, RM, WA and CL were involved and made comments during the drafting of the manuscript. All authors read and approved the final manuscript.
